# Wernicke Encephalopathy Caused by Avoidance-Restrictive Food Intake Disorder in a Child: A Case-Based Review

**DOI:** 10.3390/diseases12060112

**Published:** 2024-05-24

**Authors:** Ida Turrini, Clotilde Guidetti, Ilaria Contaldo, Silvia Pulitanò, Donato Rigante, Chiara Veredice

**Affiliations:** 1 Pediatric Neurology Unit, Policlinico Universitario A. Gemelli IRCCS, 00168 Rome, Italy; ida.turrini@guest.policlinicogemelli.it (I.T.); chiara.veredice@policlinicogemelli.it (C.V.); 2 Pediatric Intensive Care Unit, Policlinico Universitario A. Gemelli IRCCS, 00168 Rome, Italy; 3 Università Cattolica Sacro Cuore, 00168 Rome, Italy; 4 Department of Life Sciences and Public Health, Policlinico Universitario A. Gemelli IRCCS, 00168 Rome, Italy

**Keywords:** Wernicke encephalopathy, avoidance-restrictive food intake disorder, thiamine, personalized medicine, cognitive behavioral therapy, aripiprazole, child

## Abstract

Background: Wernicke encephalopathy (WE) is an acute and potentially fatal neuropsychiatric disorder resulting from thiamine deficiency: its etiology and clinical presentation can be heterogeneous and arduously recognized, especially in children and adolescents. Case presentation: An 8-year-old girl arrived to the emergency room with ataxic gait, nystagmus, and mental confusion after a 10-day history of repeated severe vomiting; her recent clinical history was characterized by restricted nutrition due to a choking phobia, which caused substantial *weight loss*. Brain magnetic resonance imaging revealed a bilaterally increased T2 signal in the medial areas of the thalami and cerebral periaqueductal region. Diagnosis of WE based on clinical and neuroradiological findings was established and confirmed after labwork showing low serum thiamine. Following psychiatric evaluation, the patient was also diagnosed with avoidance-restrictive food intake disorder (ARFID), which required starting cognitive behavioral therapy and introducing aripiprazole. The patient displayed improvement of the radiological findings after one month and complete resolution of her neurological symptoms and signs. Conclusions: Eating disorders like ARFID might forerun acute signs of WE; this possibility should be considered even in pediatric patients, especially when atypical neurological pictures or feeding issues come out.

## 1. Introduction

Deficiency in several micronutrients such as vitamins and minerals has been described in many patients with an eating disorder (ED) [1], and in particular, a severe deficiency in thiamine (vitamin B1) can be associated with a peculiar neuropsychiatric syndrome named Wernicke encephalopathy (WE). This is a rare neurologic emergency due to a single vitamin deficiency, thiamine, which might have a poor outcome if brain thiamine deficiency is left uncorrected: the vast majority of patients with WE are adult patients who are alcoholics. Indeed, many clinicians might miss recognizing WE in nonalcoholic individuals or in patients at risk for developing thiamine deficiency [2]. WE was first described in 1881 by Carl Wernicke, a German psychiatrist, in association with autopsy findings of punctate hemorrhages in close proximity to the third and fourth ventricles and cerebral aqueduct. Such a diagnosis is clinically based on the typical “triad” of ophthalmoplegia, gait ataxia, and cognitive impairment, consisting of confusion, delirium, and memory disturbance with body temperature dysregulation and hypotension. The majority of affected patients may have a history of chronic alcohol abuse, but they might simply display undernutrition caused by famine, malabsorption, and increased metabolic requirement or inadequate intake of vitamins, including thiamine [1,2].

Thiamine is one of the water-soluble B vitamins, naturally present in foods such as whole grains, cereals, legumes and meat, which can be added to some nutritional products, being available as a dietary supplement. It is crucial in different biochemical pathways related to energy metabolism and, therefore, decisive for growth, development, and regular function of cells. Without thiamine, glucose is metabolized through less efficient anaerobic pathways that produce lactic acid. Acidosis caused by thiamine deficiency can profoundly affect the central nervous system, mostly the thalami, mammillary bodies, oculomotor nuclei, and cerebellar vermis, resulting in severe neuropsychiatric morbidities [3,4]. Humans have the power to store thiamine primarily in the liver; however, a continuous thiamine supply from the diet is necessary, as thiamine body storage can be depleted in as little as 4 weeks, warranting central nervous system vulnerability related to thiamine critical contribution to numerous brain metabolic activities [5].

In 2013, avoidant/restrictive food intake disorder (ARFID) was first introduced as a new ED in the Diagnostic and Statistical Manual of Mental Disorders (DSM)-5 [6]. This feeding disturbance is characterized by either avoidant or restrictive nutritional habits, leading to abstaining from eating foods, which usually results in one or more of the following: progressive weight loss or failure to achieve an expected weight gain, nutritional deficiencies, dependence on either enteral feeding or oral nutritional supplements, and interference with regular and normal psychosocial functions [7]. The DSM-5 describes three core ARFID presentations: (a) food avoidance based on the sensory characteristics of foods, (b) lack of interest in different foods or in eating specific foods, and (c) fear of the adverse consequences associated with food intake, mostly after any traumatic events associated with eating, e.g., choking or uncontainable vomiting. Unlike anorexia nervosa and bulimia nervosa, ARFID is neither characterized by any preoccupation about self-body shape and body weight, nor by “intentional” behaviors aimed at achieving weight loss [8,9]. 

So far, the association of WE and thiamine deficiency in relationship with EDs has been mainly reported for patients with anorexia nervosa [10,11,12,13,14]. Our report is related to a young girl presenting with WE and a history of ARFID, who showed improvement after starting thiamine supplementation combined with cognitive behavioral therapy (CBT) and aripiprazole. A revision of the medical literature on this peculiar disease occurring in childhood has been provided herein.

## 2. Case Presentation

An 8-year-old girl was admitted to the Pediatric Intensive Care Unit in the Policlinico A. Gemelli IRCCS, Rome (Italy), due to mental confusion, ataxic gait, nystagmus, and uncontrollable vomiting. When first evaluated, the child had a weight of 30 kg (64th percentile), her height was 130 cm (39th percentile), and her body mass index was 17.8 kg/m^2^. The patient’s diet had been out of control and largely based on high-carbohydrate foods before this hospitalization. In particular, two months before, she presented with accidental choking while eating a solid meal. After this episode, the girl started to progressively restrict her diet in terms of both quantity and number of meals, developing a weight loss of 12 kg over that period of time; in addition, her parents reported that ten days earlier, she had presented with recurrent severe vomiting, but outpatient esophagogastroduodenoscopy had showed no abnormalities.

**Figure 1:**
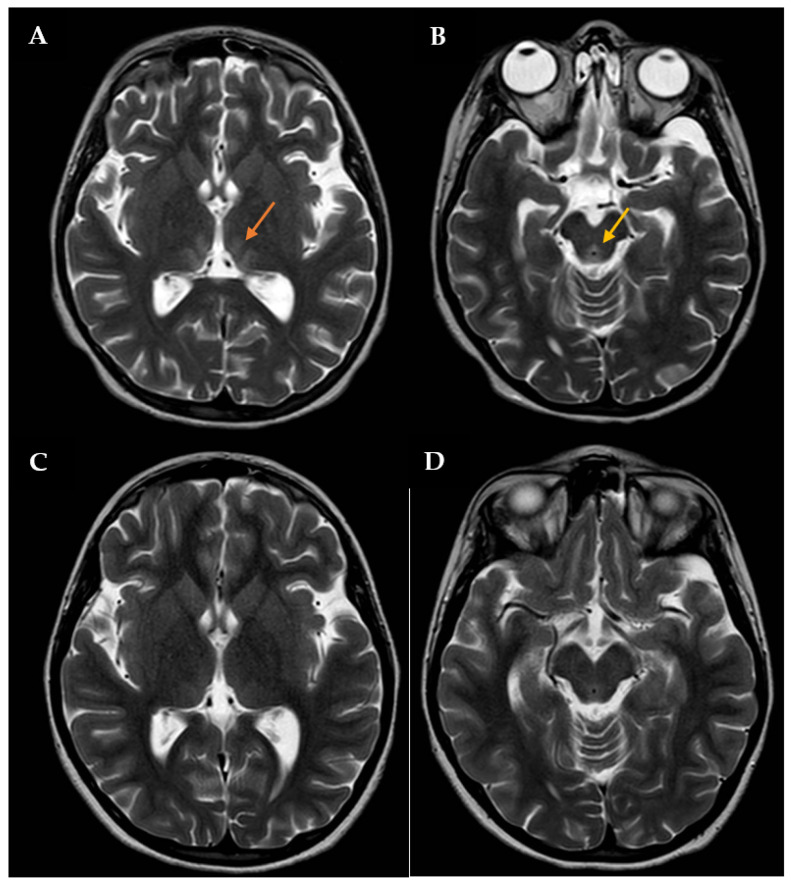
Brain MRI with contrast showing T2 signal hyperintensity involving the medial thalami (orange arrow in **A**) and the periaqueductal region (yellow arrow in **B**). The patient was initially treated with high-dose intravenous thiamine, then with oral thiamine on a daily basis. A new brain MRI showing a significant improvement in lesions was assessed one month after disease onset (**C**,**D**).

During this hospitalization, the patient’s condition actually deteriorated, requiring endotracheal intubation and intermittent positive pressure ventilation. The results of routine hematologic and biochemical measurements, including glucose, sodium, potassium, calcium, urea, creatinine, amylase, lipase, alanine aminotransferase, aspartate aminotransferase, gamma-glutamyl transpeptidase, alkaline phosphatase, and blood coagulation parameters, were all within normal limits. Cerebrospinal fluid analysis was also normal. The search for infectious agents, including human immunodeficiency virus and different neurotropic viruses, was completely negative. To rule out the autoimmune nature of such conditions, anti-nuclear, anti-DNAds, anti-onconeural, anti-aquaporine-4, anti-myelin oligodendrocyte glycoprotein, anti-myelin associated glycoprotein, and anti-ganglioside antibodies were also measured, with all being negative. An expanded screening for metabolic diseases and endocrinopathies was performed, with negative results. Brain computed tomography (CT) scan revealed low-density signal abnormalities in the paraventricular thalamic regions, while brain magnetic resonance imaging (MRI, Figure 1A,B) showed slight bilateral symmetrical T2 hyperintensity in the medial thalamic nuclei, along the walls of the third ventricle and in the periaqueductal region. Neuroradiological and clinical signs were consistent with WE. Intravenous therapy with thiamine was subsequently started (600 mg twice daily). The patient’s clinical condition improved four days after starting treatment. Therefore, the girl was extubated, and two days after, she was transferred to the Child Neuropsychiatry ward. At that time, the girl had both horizontal and vertical nystagmus, eyelid ptosis, and mild ataxic gait; she was also receiving enteral nutrition via nasogastric tube (900 kcal daily). The serum thiamine level was obtained 14 days after admission to the Pediatric Intensive Care Unit and confirmed to be low: 23 ng/mL (tested using Electro-ChemiLuminescence ImmunoAssay; normal reference: 32–95). Then, thiamine was administered per os (100 mg/day), as agreed upon with the nutritional counseling team.

Video-EEG examination showed diffuse slowing of the background electric activity. The Wechsler Intelligence Scale for Children 4th Edition (WISC-IV) revealed normal intellectual functioning with a QIT result of 87; the other results were 92 for the verbal comprehension index, 91 for the perceptual reasoning index, 74 for the processing speed index, and 103 for the working memory index. The Schedule for Affective Disorders and Schizophrenia for School-Aged Children Present and Lifetime Version (K-SADS-PL), a semi-structured interview, was separately administered to both the patient and her mother, along with a non-structured interview. During the interview, the girl confirmed that in the previous two months she had been afraid of choking when swallowing different solid foods, so she felt anxious, troubled, and scared of eating normally. There was no evidence of body image or body weight concerns and no previous history of panic attacks. Through observation of the patient during her hospital stay and through the mother’s report, it was established that the restricted food intake was caused by the primitive fear of swallowing food; the patient met the criteria for diagnosis of ARFID, as defined by the DSM-5, and for subthreshold anxiety symptoms (phobia type). Therefore, 2.5 mg of aripiprazole given orally once daily was started. During the second week at the ward, the patient turned out to be fully able to tolerate oral foods, and enteral feeding was discontinued. In addition, at this time, serum thiamine reached a normal level (41.3 ng/mL).

The patient had an almost complete resolution of her neurological symptoms, with persistence of mild nystagmus occurring only in extreme positions of the gaze and slight instability in static and dynamic equilibrium tests; the video-EEG revealed a normal organization of the patient’s cerebral electrical activity. She was discharged on 100 mg of oral thiamine and oral aripiprazole (increased to 2.5 mg twice a day), while outpatient CBT was also arranged. After approximately 1 month of thiamine therapy, a brain MRI showed a full resolution of the previously reported altered signals in the thalami along the walls of the third ventricle and periaqueductal areas (Figure 1C,D).

While staying at home, the girl suffered from sporadic episodes of esophageal food bolus impaction followed by vomiting, and the choking phobia was sporadically reported in spite of regular CBT sessions. The patient’s family was supported by community social services with lifestyle education programs targeting the child’s diet quality improvement. Oral thiamine supplementation was maintained for an overall period of 14 months. Treatment with aripiprazole is still ongoing, and neuropsychiatric monitoring is performed on a biannual basis. Nevertheless, the more recent issue in this girl has been characterized by overnutrition with high-calorie nutrients, revealing a persistent conflict between the patient and food: her last weight (at 10 years) was 53 kg (99th percentile), her height was 139.5 cm (80th percentile), and her body mass index was 27.2 kg/m^2^.

## 3. Discussion and Case-Based Review of the Medical Literature

WE is an acute but reversible brain disorder induced by thiamine deficiency, traditionally related to chronic alcoholism. However, chronic alcohol consumption does not necessarily result in WE if dietary thiamine intake is adequate: in fact, thiamine deficiency may be induced by several additional potential mechanisms, such as genetic predisposition, impaired absorption of thiamine from the gut, impairment of storage in the liver or in other tissues, thiamine transport abnormalities, decreased phosphorylation to thiamine pyrophosphate, or excessive requirements for the metabolism of alcohol. In addition, multiple clinical sceneries can exacerbate WE, such as diet imbalance, prolonged intravenous nutrition, anorexia nervosa, and malabsorption. Diagnosis of thiamine deficiency may be delayed in nonalcoholic patients as a result, and the early stages of deficiency may be heralded by gastrointestinal abnormalities such as slow gastric emptying. Many clinicians may underestimate the risk of nonalcoholic WE, in which sudden and severe weight loss or vomiting can be precociously encountered [15]. The traditional triad of WE includes ophthalmoplegia, ataxic gait, and impaired mental status, though these three signs are uncommonly found together [13]. Even if diagnosis of WE is made at a clinical level, systematic reviews suggest that Caine’s criteria, using one symptom of the triad in combination with malnutrition, might have a higher sensitivity than the complete triad for the recognition of WE [14], requiring that dietary deficiencies are combined with oculomotor abnormalities, cerebellar dysfunction, or cognitive impairment. Even if thiamine absolute deficiency may directly cause WE, diagnosis may sometimes require the support of brain imaging, revealing symmetric signal abnormalities with diffusion restriction and lack of normal enhancement in the bilateral thalami or changes in the mammillary bodies. However, though imaging studies and labwork with low serum thiamine levels may support the diagnosis of WE, these findings alone are not diagnostic. Furthermore, if cases of WE are not identified, patients’ neurologic status may worsen, even leading to death. One of the major barriers to early diagnosis of WE is the low index of suspicion in nonalcoholic patients, mostly if young patients with anorexia nervosa, patients on dialysis, or patients undergoing a long-lasting parenteral nutrition [4].

Foods rich in thiamine include whole grains, fortified or enriched grains, brown rice, soybean seeds, dried beans or peas, infant formulas, and meats; thiamine is physiologically absorbed from the duodenum into the blood stream, but small amounts of thiamine can be stored in the liver, though for a limited period of time [4]. In particular, thiamine diphosphate acts as a cofactor for a number of thiamine-dependent enzymes, and different metabolic pathways are involved, including production of adenosine triphosphate and oxidative degradation of carbohydrates, fatty acids, and amino acids. Without thiamine, pyruvate is unable to enter the tricarboxylic acid cycle, which leads to conversion of pyruvate into lactic acid; in addition, thiamine acts as a coenzyme for transketolase in the pentose phosphate pathway. Because thiamine-dependent enzymes have an irreplaceable role in cerebral energy utilization, thiamine deficiency may propagate brain tissue injury by inhibiting metabolism in further regions with higher metabolic demands or higher thiamine turnover, with decreased energy resources and selective neuronal death. In the end, the reduced production of succinate involved in gamma-aminobutyric acid metabolism might also impact neuronal excitability [4].

A multitude of complications may follow thiamine deficiency, including cardiomyopathy (“wet beriberi”), distal neuropathy (“dry beriberi”), central pontine myelinolysis, and a large spectrum of disorders, including WE and Wernicke–Korsakoff syndrome. The administration of thiamine can naturally lead to improvement of ocular dysfunction, gait abnormality, and disrupted state of consciousness, as well as more complex signs such as bradypsychia and bradylalia, which are openly referred to WE [16]. The Wernicke–Korsakoff syndrome is characterized by chronic neuropsychiatric symptoms occurring as a consequence of WE: its detection is pivotal to halt the progression of WE. Other causes of WE in adults may be related to hyperemesis gravidarum, intestinal obstruction malignancy, or bariatric surgery (Roux-en-Y gastric bypass, sleeve gastrectomy, duodenal switch, etc.), while the conditions associated with WE in childhood are nutrient deprivation, primitive gastrointestinal disorders, prolonged infectious illnesses including tuberculosis, acquired immunodeficiency syndrome, nephrotic syndrome, and end-stage renal diseases requiring dialysis and prolonged parenteral nutrition without a proper supplementation of thiamine [17].

The recognition of symmetrical hypodense lesions in the basal ganglia and medial thalami/hypothalamic/subthalamic areas on CT scans of the brain is a clue to consider WE in at-risk young patients [18]. Common brain MRI findings encompass symmetric T2 hyperintensities in the thalamus, mammillary bodies, and periaqueductal gray matter with restricted diffusion and prominent lactate peak, all findings that may suggest WE, though these findings cannot be evocative for diagnosis without an appropriate clinical scenery [19]. There are few data about children with WE, with most cases being misdiagnosed; identifying neurologic signs, combined with imaging studies such as brain CT or MRI, should be essential for a precocious suggestion of WE. It is important to highlight that blurred vision and nystagmus may predominate over further manifestations in children with WE, in combination with vomiting, poor appetite, lethargy, and weight loss [20]. A brain MRI can help diagnose WE in children, following hyperintense signal in specific districts that are more prone to display sensitivity to thiamine deficiency, such as the thalamus, mammillary bodies, cerebral aqueduct, and midline cerebellum [21].

The presence of a nutritional deficiency including thiamine should always be assessed in patients displaying poor nutritional intake or intractable vomiting, and pediatric patients being excluded for having metabolic derangements should be considered for WE. Nutritional deficiencies related to EDs derive from starvation in anorexia nervosa and other psychiatric disorders [22], but a persistent disturbance leading to avoidance of foods might result in significant weight loss, giving rise to the picture of ARFID. Currently, to the best of our knowledge, only four pediatric cases of WE have been related to ARFID [23,24,25,26].

A 4-year-old boy with a history of food aversion, difficulty in swallowing, and decreased appetite was hospitalized with intractable vomiting and weakness of four days’ duration. The vomiting was ondansetron-resistant, and the child developed severe dehydration and weight loss, as well as ophthalmoplegia and gait ataxia. Extensive metabolic testing was negative. After three days, the patient became more lethargic, with bilateral ptosis, upgaze palsy, and abnormal walking. The brain MRI demonstrated that the thalamus, mammillary bodies, and periaqueductal regions were hyperintense in the diffusion-weighted images, while the serum thiamine level was severely decreased. He was ultimately found to have WE secondary to ARFID and was started on high-dose intravenous thiamine, showing an excellent response after only three days [23].

A 12-year-old girl was hospitalized for rehydration secondary to decreased oral intake in the previous three weeks due to crampy epigastric pain and post-prandial emesis. In particular, the girl had lost over 40 kg of her body weight in the past year due to self-imposed dietary restrictions, eliminating sugary foods. The patient denied inducing vomiting and binging. Her metabolic profile was within normal limits, and many radiological investigations ruled out acute abdomen and superior mesenteric artery syndrome. Ondansetron was initially given, but post-prandial vomiting persisted, while double vision was noted in combination with blurred vision, nystagmus, and unsteady gait. The brain MRI was normal, but the pediatric gastroenterologist empirically recommended thiamine supplementation as a trial for WE. She was ultimately confirmed to have severe thiamine deficiency with ARFID-related WE [24].

A 10-year-old girl had a two-month history of food refusal, vomiting, and lethargy; two months before, she had begun to reject many foods and drink only soft beverages. The neurological examination revealed confusion, bemusement, short-term memory impairment, and bilateral complete lateral and upgaze palsy. The brain MRI identified abnormally bright signals in the dorsomedial thalamic nuclei and in the posterior putamina. Treatment with thiamine was started and led to a considerable neurologic improvement after only 48 h [25].

A 10-year-old boy was hospitalized after a two-month history of food refusal and weight loss, following a choking episode caused by raisin ingestion. Diplopia was the reason for requiring medical assistance. In particular, he was unable to fully abduct his eyes beyond the mid position, but he also presented nystagmus and ataxia. The presumptive diagnosis was a conversion disorder, but serum thiamine concentration was low, suggesting the diagnosis of WE which urgently required thiamine supplementation. Within 12 h an improvement was noted, and within 48 h he was able to abduct each eye. One month later he showed a near-complete recovery of all neurological signs [26].

None of these patients had received a psychiatric diagnosis prior to the onset of WE, and no psychological treatment had been suggested for their previous ED. All these observations highlight the importance of a complete familiar history, including eating habits, in order to evaluate the possibility of malnutrition, even with overweight patients.

ARFID should be considered in patients with weight loss who do not meet the criteria for anorexia or disordered eating behaviors and distorted attitudes towards food, while the Caine criteria could be employed for testing the possibility of WE and immediately starting treatment with thiamine. Based on the DSM-V, ARFID may be disclosed by significant weight loss, nutritional flaws, requirement of enteral feeding, and unwieldy psychosocial dysfunction [22,23,24,25,26,27]. Due to the broad differences in age, developmental stage, gender, underpinning mechanisms, and clinical presentation, individuals with ARFID require different specialist skills and various interventions from a range of clinical services and professions, including pediatrics, pediatric emergency care, pediatric endocrinology, dietetics, pediatric gastroenterology, child and adolescent psychiatry, general mental health services, social welfare, and occupational therapy. Patients with ARFID who need hospitalization often have significant mental health comorbidities which might combine with adverse parental feeding styles [28]. Also, pediatricians should take into account WE in children with ARFID, who show neurological symptoms deriving from thiamine deficiency related to loss of interest for food and/or fear of adverse consequences after eating. Unfortunately, there is no consensus about the most exact dosage, frequency, and overall duration of thiamine administration in these patients. Many experts suggest treating WE with high parenteral doses of thiamine and gradually reducing them after both clinical and radiological improvement, as any accumulated damage in the central nervous system makes the use of oral thiamine therapeutically inadequate. Thiamine supplementation should be initiated at the earliest suspicion of thiamine depletion, even before laboratory confirmation is available [29].

Our present report is related to an 8-year-old girl with a rapidly deteriorating neurological syndrome consistent with WE caused by new-onset ARFID: this patient did not present the classic triad of WE, but otherwise, she met all the criteria suggested by Caine. Without a doubt, the most reliable method to establish thiamine deficiency is measuring erythrocyte transketolase activity (at baseline and after addition of thiamine pyrophosphate). Fortunately, the timely administration of thiamine allowed for the resolution of the patient’s neurological signs, while CBT associated with aripiprazole allowed a progressively adequate control of the patient’s symptoms. In recent years, clinicians’ awareness of ARFID has enabled advocacy for more research and specific development of treatments for this condition. The medical literature about treatment of ARFID in adults consists of single patient case studies and small case series [30]. Due to the recent development of ARFID diagnostic criteria and due to the considerable variability in presentation, a wide array of management approaches have been proposed, including CBT, psychotherapy, and psychotropic medications. These approaches have been tested in outpatient settings, but findings related to efficacy are limited in consideration of the small number of studies available.

Our patient was treated with aripiprazole, an atypical antipsychotic drug acting as a dopaminergic antagonist or stabilizer with serotonergic activities, which is largely used for the treatment of schizophrenia and bipolar disorders, but also as an off-label tool for attention-deficit hyperactivity disorder, insomnia, obsessive-compulsive disorders, personality disorders, post-traumatic stress disorders, substance abuse disorders, Tourette’s syndrome, and EDs [31]. Common side effects of atypical antipsychotics are akathisia, hyperphagia, weight gain, and diabetes mellitus, but—among all atypical antipsychotics—aripiprazole is generally thought to have fewer effects on body weight gain [32].

## 4. Conclusions

Thiamine deficiency is a potential risk in any chronically malnourished individual, but it is rather rare in children, in whom it may represent the foundation of acute neurological signs, which might herald WE. The presence of nutritional deficiencies, including thiamine, should always be considered in patients with a history of nutritional issues and intractable vomiting. Pediatric patients ruled out for metabolic derangements should be considered for a diagnosis of WE. Both early recognition and treatment of thiamine deficiency may slow down the progression to WE and allow a better outcome for the neurological signs. Specifically, ARFID should be recognized for its potential to cause deficiencies in vitamins, and WE should be considered if a pediatric patient shows a rapidly deteriorating neurological syndrome. Our report confirms that thiamine deficiency caused by ARFID in a child may lead to WE and that thiamine supplementation is crucial for recovery.

## Data Availability

No new data were created.
